# A Systematic Review of Experimental and Clinical Acupuncture in Chemotherapy-Induced Peripheral Neuropathy

**DOI:** 10.1155/2013/516916

**Published:** 2013-07-24

**Authors:** Giovanna Franconi, Luigi Manni, Sven Schröder, Paolo Marchetti, Nicola Robinson

**Affiliations:** ^1^Department of Systems Medicine, Tor Vergata University, 00133 Rome, Italy; ^2^Institute of Translational Pharmacology, National Research Council, 00133 Rome, Italy; ^3^HanseMerkur Center for Traditional Chinese Medicine at the University Medical Center Hamburg-Eppendorf, Martinistrasse 52, 20246 Hamburg, Germany; ^4^Sant'Andrea Hospital, Sapienza University, 00189 Rome, Italy; ^5^IDI-IRCCS, 00167 Rome, Italy; ^6^Faculty of Health and Social Care, London South Bank University, 103 Borough Road, London SE1 0AA, UK

## Abstract

Chemotherapy-induced peripheral neuropathy (CIPN) is a common side effect that can be very disabling and can limit or delay the dose of chemotherapy that can be administered. Acupuncture may be effective for treating peripheral neuropathy. The aim of this study was to review the available literature on the use of acupuncture for CIPN. The systematic literature search was performed using MEDLINE, Google Scholar, Cochrane Database, CINHAL, and ISI Proceedings. Hand searching was conducted, and consensus was reached on all extracted data. Only papers in the English language were included, irrespective of study design. From 3989 retrieved papers, 8 relevant papers were identified. One was an experimental study which showed that electroacupuncture suppressed CIPN pain in rats. In addition, there were 7 very heterogeneous clinical studies, 1 controlled randomised study using auricular acupuncture, 2 randomized controlled studies using somatic acupuncture, and 3 case series/case reports which suggested a positive effect of acupuncture in CIPN. *Conclusions*. Only one controlled randomised study demonstrated that acupuncture may be beneficial for CIPN. All the clinical studies reviewed had important methodological limitations. Further studies with robust methodology are needed to demonstrate the role of acupuncture for treating CIPN resulting from cancer treatment.

## 1. Introduction

Chemotherapy-induced peripheral neuropathy (CIPN) is a common side effect after patient exposure to chemotherapy agents such as vinca alkaloids, platinum derivatives, and taxanes and to newer agents such as bortezomib and thalidomide. CIPN is the second most important side effect in frequency after hematologic toxicity and can limit or delay the dose of chemotherapy that can be administered [1]. Sensory, motor, and/or autonomic neurotoxicity can be very disabling and/or painful, creating a major impact on patients' quality of life and adherence to treatment.

Conventionally, CIPN is prevented with the use of intravenous calcium and magnesium, without reducing treatment response. Pharmacological treatment of other CIPN symptoms like numbness or palsies is not usually effective, but there are some other options for treatment of neuropathic pain such as the use of antiepileptic and tricyclic antidepressant drugs (e.g., carbamazepine, lamotrigine, gabapentine or pregabalin, and venflaxine). There is no established pharmacological treatment for neuroregeneration [2, 3].

The mechanism of neurotoxicity of the antineoplastic agents is unclear. CIPN generally arises as a consequence of the disruption of axoplasmic microtubule mediated transport, distal axonal degeneration, and direct damage to the sensory nerve cell bodies of the dorsal root ganglia (DRG). Mitochondrial damage in the DRG neuron has been described as well [4]. The DRG neurons lack a vascular barrier, in contrast to those in the central nervous system, and are more exposed to the neurotoxic effects of the antineoplastic agents. Both central and peripheral mechanisms seem to be involved. 

Acupuncture stimulates areas of the skin using different methods including the insertion of thin needles that are then manipulated manually or electrically. In animal models acupuncture has been shown to reduce neuropathic pain in a variety of experimental conditions. Cold allodynia has been reduced in rats treated with electroacupuncture through the mediation of spinal alfa2-adrenergic receptors [5], while in mechanical allodynia electroacupuncture has been shown to act through mu and delta but not kappa opioid receptors [6]. Another possible effect of electroacupuncture in an experimental model of neuropathy may be the inhibition of COX2 expression [7]. It has been shown that electroacupuncture at the acupoint Zusanli (ST36) reduced the mechanical allodynia in a neuropathic model, normalising the expression profile of hypothalamic proteins, that have been mainly identified as being involved in inflammatory processes, metabolism and signal transduction [8]. Ko and colleagues [9] examined the mechanism of neuropathic pain and the analgesic effects of acupuncture at the molecular level by cDNA microarray analysis. They observed that the expression of 68 genes had more than doubled in a model of neuropathic pain in the rat but returned to normal after treatment with electroacupuncture. The genes were involved in biological processes such as signal translation, gene expression, and nociceptive pathways [9]. Also the expression of the sigma opioid receptor was decreased by 50% in the neuropathic pain model and was returned to normal after acupuncture. This could explain the poor response of neuropathic pain to the treatment with opioids, since the opioid receptor is downregulated in this condition, and could partially explain the analgesic action of acupuncture in neuropathic pain, because acupuncture normalises the opioid receptor expression and also increases the release of endogenous opioid peptides. 

Cancer patients often seek additional help for their disease or for treatment-related side effects. A European survey in 13 countries [10] showed a prevalence of 35.9% of complementary therapy use (range among countries 14.8% to 73.1%). Acupuncture was used by 3.9% patients before cancer diagnosis and by 3% patients after cancer diagnosis. Acupuncture has also been shown to be effective for chemotherapy induced nausea and vomiting [11], xerostomia induced by radiation therapy [12, 13], fatigue [14], anxiety, depression, and insomnia [15]. Furthermore, acupuncture has been used encouragingly to treat peripheral neuropathy associated with diabetes and HIV [16–22].

In this paper the existing evidence of acupuncture effectiveness and/or efficacy for CIPN has been systematically reviewed.

## 2. Methodology

To review the existing clinical and experimental studies of acupuncture in CIPN, the systematic literature search was performed from the databases inception up until January 2012 using MEDLINE, Google Scholar, Cochrane Database, CINHAL (Cumulative Index to Nursing and Allied Health Literature), CNKI (China National Knowledge Infrastructure), Wanfang Med Online, and ISI Proceedings for conference abstracts. The keywords searched were (acupoint* OR acupuncture OR electro-acupuncture OR electroacupuncture OR moxibustion) AND “peripheral neuropathy.” The CINHAL, CNKI, and Wanfang Med Online Databases did not allow logical searches with AND, so we used simple combinations of the search words. Historical searches of reference lists of relevant articles were also undertaken. To be included in the review, a study had to explore either the efficacy or the effectiveness of acupuncture needling for CIPN in either human or animal models irrespective of design. All papers with at least an abstract in English were included. Study selection was performed by two reviewers (Giovanna Franconi and Luigi Manni) with disagreement resolved by discussion and adjudication.

## 3. Results

A total of 3989 articles were retrieved from electronic searches and subsequent examination of reference lists of the clinical and review articles. After screening titles and/or abstracts, 3891 articles were excluded for the following reasons: the focus was on an intervention other than acupuncture, the neuropathy was not related to chemotherapy, the acupuncture treatment plan included additional interventions/modalities that were not acupuncture related, there were duplicated studies, or they were not relevant. From a total of 98 articles which were retrieved for detailed evaluation, 7 clinical studies and 1 experimental study were included in the review. For a summary of the clinical studies see Table 1.

Only one study was identified that addressed the topic of electroacupuncture (EA) and its effects on CIPN in an animal model. Meng and colleagues [30] demonstrated that EA at both low (10 Hz) and high (100 Hz) frequencies was able to improve neuropathic pain in paclitaxel-treated rats. The authors reported that low-frequency EA was more effective than high-frequency EA in relieving neuropathic symptoms and that opioid receptors antagonists (all types) abolished EA effects. It is known that low-frequency (2–15 Hz) EA engages centrally mediated endorphin, enkephalin, serotonergic, and noradrenergic analgesia, while high frequency EA (100 Hz) engages segmental-spinal opioids (dynorphin, enkephalin) and nonopioid (gamma aminobutyric acid, glycine) analgesia [31]. Thus it is conceivable that central-mediated effects of acupuncture are involved in suppression of neuropathic pain in CIPN.

For a summary of the clinical studies see Table 1. 

The first published clinical study that explored the efficacy of acupuncture in cancer pain used auricular acupuncture in a population of 90 patients with neuropathic pain (despite stable medication) [23]. A small minority of patients also presented nociceptive pain. The patients were randomly divided into 3 arms: one arm with steel implants on auricular points eliciting an electrical response and 2 placebo arms with either steel implants or vaccaria seeds on auricular points not eliciting an electrical response. Patients were treated for 2 one-month cycles. After 2 months the pain VAS score was significantly decreased in the true acupuncture group, while there was no effect of placebo.

In one prospective case series 5 patients were treated with manual acupuncture for their chemotherapy-induced neuropathy [24]. Acupuncture was performed once a week according to TCM diagnosis using acupoints CV6 (Qihai), ST36 (Zusanli), LI11 (Quchi), EX-LE10 (Bafeng), and EX-UE9 (Baxie) for two 6-week cycles, separated by 4 weeks. All patients showed an improvement in pain score and on the WHO grade of neuropathy after treatment with acupuncture. There were no observed side effects, and benefits persisted for 6 months of followup in 4/5 patients.

Xu et al. [25] studied 64 patients with CIPN induced by paclitaxel or oxaliplatin. The patients were randomized to an acupuncture group or a control group treated with cobamamide. The acupuncture treatment included points such as Hegu (LI4), Taichong (LR3), Zusanli (ST36), Qihai (CV6), and Quchi (LI11) and was performed for an unspecified length of time. The outcome was an evaluation of neurotoxicity assessed by a CIPN questionnaire. The twenty patients in the acupuncture group significantly improved, compared to the 12 patients in the control group.

A case report [26] described one patient with multiple myeloma and bortezomib-induced CIPN who was treated with 6 weekly sessions of acupuncture, followed by subsequent 8 sessions over the next 5.5 months. The treatment protocol included body acupuncture at LI4 (Hegu), TE5 (Waiguan), LI11 (Quchi), ST40 (Fenglong), EX-LE10 (Bafeng), and auricular acupuncture at shenmen, point zero, and 2 additional points were stimulated electrically. The VAS pain score decreased from 8/10 to 2/10 after 6 treatments, and the pain medication with morphine sulphate and oxycodone was stopped after 14 treatments. The patient remained pain-free for at least one year. There were no observed side effects.

A retrospective case series examined 18 patients affected by CIPN [27]. Patients were treated by 6 weekly acupuncture sessions, with acupoints selected on the basis of patient presentation at each session. No validated questionnaires were used, and side effects were not recorded. The most commonly used points were SP6 (Sanyinjiao) and ST36 (Zusanli), followed by LV3 (Taichong). After 6 weeks 82% (*n* = 14) patients reported an improvement of their neuropathy symptoms, 18% (*n* = 3) reported no change.

In a pilot controlled nonrandomised study [28], 6 patients with CIPN accepted acupuncture treatment, and 5 patients with CIPN who had refused acupuncture treatment served as controls. All patients received the best medical care, and the 6 patients in the acupuncture group were also treated with 10 weekly acupuncture treatments with a fixed protocol (ST34 Liangqiu, EX-LE12 Qiduan, and EX-LE10 Bafeng bilaterally). Nerve conduction studies (NCS) were done to confirm the presence of CIPN at baseline and 6 months later, that is, 3 months after the end of acupuncture treatment. Acupuncture significantly improved nerve conduction velocity and mean amplitude of NCS in treated patients, while there was no difference after the same time in the control group. There were no observed side effects.

The last study included in this review was a randomized controlled trial of 76 patients with gastrointestinal cancer and CIPN induced by oxaliplatin [29]. The intervention group received warm acupuncture and moxibustion, and the control group was treated with Neurotropin 4 mg given intramuscularly every day for 21 days. The intervention receiving acupuncture and moxibustion reported a significantly improved quality of life and reduction in neurotoxic symptoms.

## 4. Discussion

Complementary therapies in cancer care are used primarily to treat the symptoms associated with cancer and its treatments. This review suggests that although there are some indications that acupuncture may be effective in improving symptoms and neural damage associated with CIPN, the current evidence available is limited.

The positive effects of acupuncture in CIPN consist in a reduction in the pain score in most studies. Pain is the most common and the best studied indication for acupuncture, and acupuncture has been recommended as a complementary therapy for pain control or for reducing the amount of pain medicine in cancer patients. According to the evidence-based guidelines of the American College of Chest Physicians for lung cancer [32], acupuncture is recommended as a complementary therapy for lung cancer when pain is poorly controlled or when side effects such as neuropathy or xerostomia are clinically significant (grade 1A recommendation). The rationale is based on the analgesic action of acupuncture in acute and chronic pain and in cancer pain. Furthermore, studies on pain using functional magnetic resonance (fMRI) showed that acupuncture could modulate the cognitive-affective aspects of pain perception [33].

Improvement was also reported for other symptoms of CIPN in the paper by Wong and Sagar [24], where the effects of acupuncture were measured by the WHO CIPN score, which takes into account both the sensory and motor abnormalities of CIPN. One study [28] evaluated acupuncture effects with nerve conduction studies, which allowed a separate measurement of motor and sensory signals and showed a significant positive effect of acupuncture on motor and sensory parameters.

The studies included in this systematic review were very heterogeneous: 3 studies [23, 25, 29] were prospective randomized controlled trials, while another [28] was a retrospective analysis of a controlled study. These controlled studies showed a specific effect of acupuncture, unrelated to skin penetration. The remaining studies were uncontrolled case reports or case series. Such uncontrolled studies may present bias and lead to false positive results. The issue of choosing a control in acupuncture research is not a simple one, as placebo/sham acupuncture shares many pathways with true acupuncture (i.e., activation of opioid system as well as other pain-controlling neurotransmitters systems and activation of cerebral areas on fMRI), and the placebo/sham acupuncture used in acupuncture studies is not necessarily inert [34–37].

Different protocols were utilized to treat CIPN: auricular acupuncture only, and somatic acupuncture only, combined auricular and body acupuncture, each applied on different combinations of acupoints. Acupuncture protocols are usually standardized in acupuncture research, but this may not reflect what clinical acupuncturists do every day in their clinics, as acupuncture in TCM is a very individualized medicine [38]. Furthermore the choice of acupuncture points in a protocol depends on the reference system, which comprises many different schools and different approaches to acupuncture, such as acupuncture according to traditional Chinese medicine, medical acupuncture, Japanese acupuncture, French auricular acupuncture, trigger-point acupuncture, acupressure, electroacupuncture, and transcutaneous electrical nerve stimulation (TENS) of acupuncture points, among others [39], each one with a different approach to comparable problems. Future studies with sufficient number of patients should also address the issue of whether a pragmatic approach or a protocol approach should be employed.

Heterogeneity was also present when considering the outcome measurements, which ranged from subjective evaluation to pain VAS score to nerve conduction studies (NCS), which make it impossible to compare studies. More objective outcome measurements are advisable, and among them NCS which measures the number and conduction velocities of large myelinated fibers and relates to both the clinical subjective improvement and the histological nerve healing.

**Figure 1:**
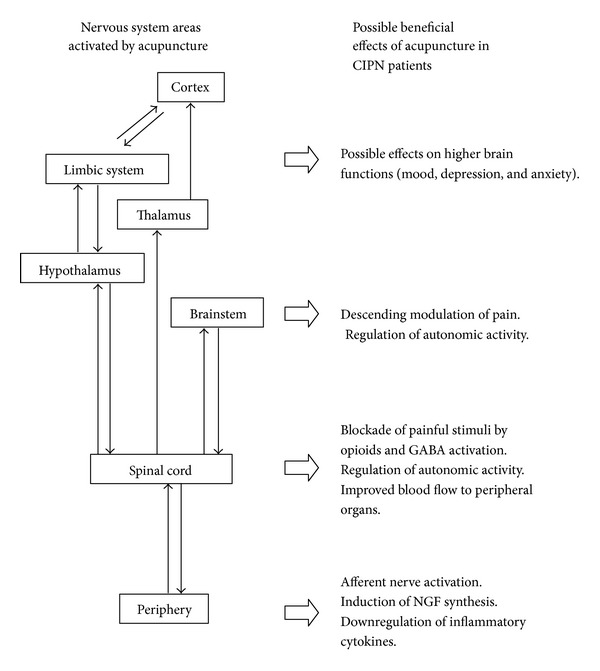
Possible targets of acupuncture treatment in CIPN. On the left side are the nervous system areas which are activated by acupuncture; on the right side are some possible beneficial effects of acupuncture in CIPN patients. CIPN = chemotherapy-induced peripheral neuropathy; GABA = gamma amino butyric acid; NGF = nerve growth factor.

Neuronal damage by antineoplastic agents probably activates second messenger systems which cause hyperalgesia, allodynia, and pain, because it may be relieved by supplementation with trophic factors such as NGF, insulin growth factor 1 (IGF-1), and neurotrophin 3 (NT-3) [40]. There is a large experimental evidence base on the involvement of NGF in CIPN [41–44]. NGF promotes physiological maturation, survival, and expression of the specific phenotype in primary sensory neurons located in the DRG [45]. Acupuncture analgesia is an effect that has been amply demonstrated and occurs via the activation of different systems, involving nerves, hormones, cytokines, and other mediators [46]. At a neuroendocrine level acupuncture modulates various neurotrophins and growth factors including NGF [47], glial-derived neurotrophic factor (GDNF) [48–50], brain-derived neurotrophic factor (BDNF) [51, 52], and insulin growth factor (IGF) [53]. It is possible that the action of acupuncture on neuropathic pain be mediated by enhancement of spinal/central GABA-ergic, serotoninergic, and adrenergic neurotransmission [54–58] as well as by the action of acupuncture on the NGF system, driving NGF signalling toward its downregulation with parallel decrease in sensory neurons hypersensitization [59]. Thus, acupuncture can modify the expression of different genes and the expression of genes that control transcriptional factors that are crucial for cell homeostasis [60]. In Figure 1, we summarized the acupuncture mechanisms and mediators in CIPN based on what we know from animal studies of diabetic neuropathy [59, 61] and from human studies of brain imaging during acupuncture [62]. 

It is interesting to note that all the studies which used somatic acupuncture and described their protocol [24, 26–28] employed local points. EX-LE10 (Bafeng) is 4 points on the instep of each foot, proximal to the margin of the webs between each two neighbouring toes, while EX-UE9 (Baxie) is 4 points proximal to the margin of the webs between each two of the five fingers of a hand. The rationale behind the choice of points located nearby or in the same dermatome of the affected limb/region might lie in the activation of spinal response after acupuncture. Indeed the western neurophysiological hypothesis on the mechanism of acupuncture efficacy proposes that needle insertion and stimulation elicits a three-level response: local (at the site of needling) that could encompass the so called “flare reaction”; segmental, that includes all the acupuncture-induced reflex variations in spinal neurotransmission, that is, GABA-ergic one; central, that refers to the overall variation induced by needle stimulation in the activity and feedback response in the brain [35]. Thus, it is possible to link the positive outcome in such studies to the spinal/segmental activation of opioids and/or GABA signalling, in accordance with previous results on animal models [30]. 

The limitations of the studies reviewed include the small sample size of most studies, the presence of poor controls or no controls, poor randomization, and lack of blinding. However, the presence of some studies of good quality which suggest a positive effect of acupuncture in CIPN support the planning of more rigorous randomised controlled clinical studies evaluating the efficacy of acupuncture in CIPN. The advantages of acupuncture are its safety and low cost, and it would be very important to demonstrate its efficacy in such a disabling and potentially dangerous side effect of cancer treatment such as CIPN.

## Figures and Tables

**Table 1:** Characteristics of the studies involving the use of acupuncture in CIPN.

Authors	Patients (*n*)	Design of the study	Intervention and control	Duration of intervention	Outcome(s)	Results
Alimi et al., 2003 [23]	90	Prospective randomized controlled trial	Auricular acupuncture versus placebo acupuncture and seeds	2 months	VAS pain score and medication consumption	True acupuncture better than placebo
Wong and Sagar, 2006 [24]	5	Prospective case series	Acupuncture (no control)	16 weeks (Two 6-week courses with a 4-week therapy free interval)	Pain score and WHO CIPN grade	Improvement
Xu et al., 2010 [25]	64	Controlled randomized trial	Acupuncture versus cobamamide	Not known	Questionnaire of peripheral neuropathy	Acupuncture better that cobamamide
Bao et al., 2011 [26]	1	Case report	Acupuncture (no control)	22 weeks	VAS pain score	No more symptoms
Donald et al., 2011 [27]	18	Retrospective case series	Acupuncture (no control)	6 weeks	Subjective symptoms	82% improved
Schroeder et al., 2012 [28]	11	Retrospective controlled nonrandomized trial	Acupuncture and best medical care versus best medical care	10 weeks	Nerve conduction studies	Acupuncture better than control
Tian et al., 2011 [29]	76	Controlled randomized trial	Warm acupuncture and moxibustion versus Neurotropin	Not known	Quality of life and neurotoxic symptoms	Acupuncture better than Neurotropin

Legend: VAS: visual analog scale; FACT-G: Functional Assessment of Cancer Therapy-General.
